# *In Vitro* Acaricidal Activity of Selected Medicinal Plants Traditionally Used against Ticks in Eastern Ethiopia

**DOI:** 10.1155/2020/7834026

**Published:** 2020-02-18

**Authors:** Jelalu Kemal, Tesfaheywet Zerihun, Sisay Alemu, Kedir Sali, Musa Nasir, Ashebr Abraha, Teka Feyera

**Affiliations:** ^1^College of Veterinary Medicine, Haramaya University, P.O. Box 138, Dire Dawa, Ethiopia; ^2^Department of Animal Science, School of Environmental and Rural Science, University of New England, Armidale, NSW 2351, Australia

## Abstract

A study was carried out to evaluate the acaricidal activities of crude methanolic extract of leaves of six medicinal plants, namely, *Vernonia amygdalina*, *Calpurnia aurea*, *Schinus molle*, *Ricinus communis*, *Croton macrostachyus*, and *Nicotiana tabacum*, against *Rhipicephalus* (*Boophilus*) *decoloratus* and *Rhipicephalus pulchellus* using an *in vitro* adult immersion test. Five graded concentrations of the crude extracts, 6.25, 12.5, 25, 50, and 100 mg/ml, were tested at different time intervals, and temporal changes in tick viability were recorded for 24 hours. Diazinon (0.1%) and distilled water were used as positive and negative controls, respectively. Standard procedures were applied to screen the phytochemical constituents of the tested plant parts. Phytochemical screening showed the presence of a condensed amount of tannins in all extracts. Starting from 30 min post exposure, the 100 mg/ml concentration of *C. aurea* and *R. communis* extracts has caused significantly higher mortality (*P* < 0.05) compared to the diazinon. A significant increase in tick mortality started 2 hr post exposure with diazinon and 50 and 100 mg/ml concentrations of *S. molle*. *Vernonia amygdalina* extract and diazinon showed a significant increase in tick mortality 3 hr post exposure with 100 mg/ml concentration. Similarly, a significant increase in tick mortality started 2 hr post exposure with diazinon and 100 mg/ml concentrations of *C. macrostachyus* and *N. tabacum*. At 24 hr post exposure, diazinon and 50 and 100 mg/ml concentrations of all the extracts have caused significantly higher tick mortality than the rest of the concentrations (*P* < 0.05). Higher concentrations of all the extracts had showed a comparable and strong acaricidal effect on *Rhipicephalus* (*Boophilus*) *decoloratus* and *Rhipicephalus pulchellus* having no significant difference with that of the positive control (*P* > 0.05) at 24 hr post exposure period. Tick killing activity of all evaluated plant extracts increases with increasing exposure time and concentration as well. Thus, all the tested plants could be used against *Rhipicephalus* (*Boophilus*) *decoloratus* and *Rhipicephalus pulchellus* as a potential alternative to substitute commercially available drugs. We recommend further study on fractionating each component separately and validating the materials.

## 1. Introduction

Ticks are destructive blood-sucking ectoparasites of livestock and wild animals causing huge economic losses, thus creating food insecurity [1], with an estimated global cost of control and productivity losses of 7 billion USD per year [2]. Their effects are diverse, including reduced growth and milk production, paralysis/toxicosis, and transmission of tick-borne diseases (TBDs) that reduce production or cause mortality [3].

Worldwide tick control is based mainly on the repeated use of acaricides, which have resulted in problems related to environmental pollution, milk and meat contamination, and the development of resistance leading to increased cost of control [4]. In Ethiopia, over the past decades, ticks are mainly controlled by using a variety of synthetic acaricides [5]. However, ticks have developed resistance against commercial acaricides in Ethiopia with the widespread, under- or overconcentrated, and frequent use of these compounds [6, 7]. Thus, there is an urgent need for new tick control strategies to overcome the drawback associated with the use of synthetic drugs. One alternative control strategy could be phytotherapy, an important component of ethnoveterinary medicine [8].

The use of natural products, mainly acaricides from the botanical source used for the control of ticks, has been the focus of research in many countries, principally to withstand the noticeable increasing frequency of acaricide-resistant tick strains. The use of botanicals for the control of ticks is compatible with traditional practices in Africa, including Ethiopia where most resource-poor farmers use plant materials to treat ectoparasites and endoparasites of livestock [2]. Acaricidal activity of crude extracts from different plants against ticks has been reported [9–13]. The phytoextracts produce acaricidal properties through diverse mechanisms: killing adult ticks, reducing tick feeding, and inhibition of egg hatching, molting, fecundity, and viability of eggs [8, 14, 15].

In far rural parts of the country including eastern Hararghe, modern veterinary drugs including synthetic acaricides are not affordable to the majority of poor livestock owners. Even when accessible, the owners tend to treat their livestock with synthetic products in a haphazard way, and misuse of chemicals illegally imported at a high cost from neighboring countries is increasing year after year. Furthermore, there is a continuous complain from the livestock keepers over the poor efficacy of most of the existing acaricidal drugs. As a result, livestock raisers continue to rely on ethnoveterinary knowledge and practices for socially acceptable, inexpensive, and locally available remedies for managing ticks and other ectoparasites affecting their livestock. Additional fact that desires due attention is that livestock owners claim a number of botanicals having acaricidal effect which need scientific evaluation by using standardized parasitological procedures.

Thus, it is quite rightly convincing that evaluating acaricidal efficacy of natural remedies can greatly contribute to control tick invasion in a cheaper and more sustainable way. Moreover, there is a great potential to develop sources of acaricides from the available medicinal plants which can be easily produced by livestock producers themselves or processed by cottage industries and used as cheap and efficient natural biocide for tick control [1]. Implementing an effective tick control strategy suitable to a specific livestock production system is a dual harvest, controlling ticks and also the TBDs. Therefore, it is necessary to undertake an acaricidal efficacy evaluation of botanicals traditionally used by the livestock owners as an alternative tick management strategy. The present work was aimed at evaluating the acaricidal activity of crude extracts of leaves of six medicinal plants traditionally used by livestock raisers as an alternative tick control strategy in eastern Hararghe, Ethiopia. The plants tested in the current biological activity assay include *Vernonia amygdalina*, *Calpurnia aurea*, *Schinus molle*, *Ricinus communis*, *Croton macrostachyus*, and *Nicotiana tabacum* leaf extracts against *Rhipicephalus (Boophilus) decoloratus* and *Rhipicephalus pulchellus* for antitick activity.

## 2. Materials and Methods

### 2.1. Study Design

This study employed an experimental study design: an *in vitro* immersion method as described by Vongkhamchanh et al. [15].

### 2.2. Collection and Identification of the Plant Materials

The plants were selected based on the scientific and ethnomedical information in the literature complemented with a preliminary ethnobotanical survey during collection from their natural habitats. The candidate plants that were included for the experiment were *V. amygdalina* (*Girawa/Dhebichaa*), *S. molle* (*Mirmir/Muka libaanataa*), *C. aurea* (*Digita/Ceekaa*), *R. communis* (*Gulo/Qobboo*), *C. macrostachyus* (*Bisana/Makannisa*), and *N. tabacum* (*Tumbahoo/Tamboo*). The selected plants were collected, identified, and verified with taxonomical studies as reported by [16]. To reduce possible contamination, especially by fungi, latex gloves were worn during leaf collection.

### 2.3. Crude Extract Preparation

Fresh leaves of the plants were separately cleaned with tap water to remove dirt and soil particles, shade dried at room temperature for two weeks, mechanically grinded, and coarsely powdered using an electric grinder. The powdered specimen was then subjected to extraction using 80% methanol by a cold maceration technique. A total of 500 g of the pounded materials was separately soaked in each extraction solvent (100 g of powder in 400 ml of solvent) followed by shaking periodically for three days and then filtered. This was repeated three times to allow the solvents extract substantial quantities of the chemical constituents from the pounded plant materials. The mixture was first filtered using gauze, and then, the filtrate was passed through sterile filter paper (Whatman No. 1, Whatman Ltd. England). Then, the filtered extract was kept overnight in a hot air oven at a temperature of 60°C to obtain the pure crude extracts. The extraction rate (%) was calculated as follows:

(1) $$\begin{array}{c} \text{Extraction rate }\left(\%\right)=\frac{\text{Weight of extracts }\left(\text{g}\right)}{\text{Weight of the plant material }\left(\text{g}\right)\text{ before extraction}}\times 100. \end{array}$$

The resulting extracts were then transferred into well-labeled vials and kept in a refrigerator until required for use.

### 2.4. Phytochemical Screening

Phytochemical screening was carried out to assess the qualitative chemical composition of crude extracts using commonly employed precipitation and coloration reaction to identify the major natural chemical groups and secondary metabolites present in the plants. Combinations of several methods were used to identify the phytochemicals of the medicinal plants. Standard screening tests were conducted using a conventional protocol and reagents on the methanolic extracts of herbs to identify the constituents as described by Sofowora [17]. The screening was done to detect the presence of bioactive principle believed to have acaricidal activities: saponins, tannins, flavonoids, steroids, phenolic compounds, alkaloids, glycosides, and triterpenes.

### 2.5. Study Parasite Collection, Transportation, and Identification

Ticks were collected from naturally infested cattle in Haramaya (Finkille, Adelle) and Harar (Erer, Dire Teyara), Eastern Ethiopia. For collection of ticks, the entire body surface of the animals was examined thoroughly, and adult ticks were collected from the body of the animals where they were available. Collected ticks were put in vials and were wrapped in cotton net gauze for oxygen supply and transported and identified in Haramaya University College of Veterinary Medicine, Parasitology Laboratory. All collected ticks from naturally infested cattle were examined under a stereomicroscope and identified according to [18].

### 2.6. Acaricidal Activity Evaluation

#### 2.6.1. Preparation of Concentrations of Crude Methanolic Extracts

The dried extracts were diluted in distilled water at the concentrations required for the bioassays (6.25 mg/ml, 12.5 mg/ml, 25 mg/ml, 50 mg/ml, and 100 mg/ml) for all tested plants. The concentrations were used for the acaricidal efficacy test. Distilled water was used as the negative control while 0.1% diazinon was used as the positive control. The positive control, 0.1% diazinon 60 EC (Adamitulu Pesticides Processing, Ethiopia), was diluted in water according to the manufacturer's recommendation (1 : 1000) before being used for further experiment [19].

#### 2.6.2. Adult Immersion Test

The *in vitro* tests were started within one hour after tick collection [19]. Ten active live adult ticks in three replications were put into the Petri dish, and 3 ml of each concentration was directly added to the three replicated Petri dishes for 2 min of exposure. After immersion, the ticks were filtered with filter paper and placed in separate Petri dishes [20]. Three millilitres of distilled water and 0.1% diazinon 60 EC were used as the negative and positive controls, respectively. The Petri dishes were incubated at 28°C with 80% relative humidity, and each tick in each Petri dish was closely observed for any death under a stereomicroscope at 30 min, 1 hr, 2 hr, 3 hr, 6 hr, 12 hr, and 24 hr time intervals [21]. The viability of ticks was checked regularly by stimulation with a needle, and ticks were recorded as dead if no reaction was shown. The percentage mortality was calculated by using a formula given by Krishnaveni and Venkatalakshmi [22] as follows:

(2) $$\begin{array}{c} \text{Mortality}\%=\frac{\text{Number of dead ticks}}{\text{Total number of ticks}}\times 100. \end{array}$$

### 2.7. Data Analysis

Collected raw data was stored in a Microsoft Excel database system used for data management. SPSS Windows version 20 was used for data analysis. Mean tick mortality and related results of the study were expressed using descriptive statistics (mean ± standard error of mean, percentage, and graph). One way analysis of variance (ANOVA) followed by Tukey's HSD multiple comparisons was used to compare differences between different *in vitro* groups. All significant levels are set at *P* < 0.05.

## 3. Result

### 3.1. Percentage Extraction Yield and Phytochemical Constituents

Table 1 summarizes the percentage extraction yields of 500 g powder of leaves of each plant. Each extract had a dark brown color and sticky and semisolid consistence. The highest and lowest percentage yields obtained were 30.67% and 12% for *R. communis* and *V. amygdalina*, respectively. The phytochemical constituents detected in the resultant crude extracts are shown in Table 2. Phytochemical screening showed the presence of condensed tannins and alkaloids in all extracts.

### 3.2. *In Vitro* Acaricidal Activity of the Plant Extracts against *Rh. decoloratus* and *pulchellus*

A significant increase in tick mortality started 3 hr post exposure with 100 mg/ml concentration of *C. aurea* extract and diazinon and 12 hr post exposure with 50 mg/ml concentration of the *C. aurea* extract. Starting from 30 min post exposure, the 100 mg/ml concentration of *C. aurea* extract has caused significantly higher mortality compared to the diazinon (*P* < 0.05). At 24 hr post exposure period, diazinon and 50 and 100 mg/ml concentrations of the extract have caused significantly higher tick mortality compared with the rest of the concentrations below 25 mg/ml (*P* < 0.05). The least concentration (6.25 mg/ml) has caused significantly higher mortality when compared with the negative control (distilled water) at 24 hr exposure time.

A significant increase in tick mortality started 2 hr post exposure with diazinon and 50 and 100 mg/ml concentrations of *S. molle* and 12 hr post exposure with 6.25, 12.5, and 25 mg/ml concentrations of *S. molle* extract. At 24 hr post exposure period, 25, 50, and 100 mg/ml concentrations of the extract and diazinon had a comparable tick killing effect compared with the rest of the concentrations below 12.5 mg/ml. However, the three higher concentrations of the extracts and diazinon had no significant difference in their effect on the parasite (*P* > 0.05).

**Figure 1:**
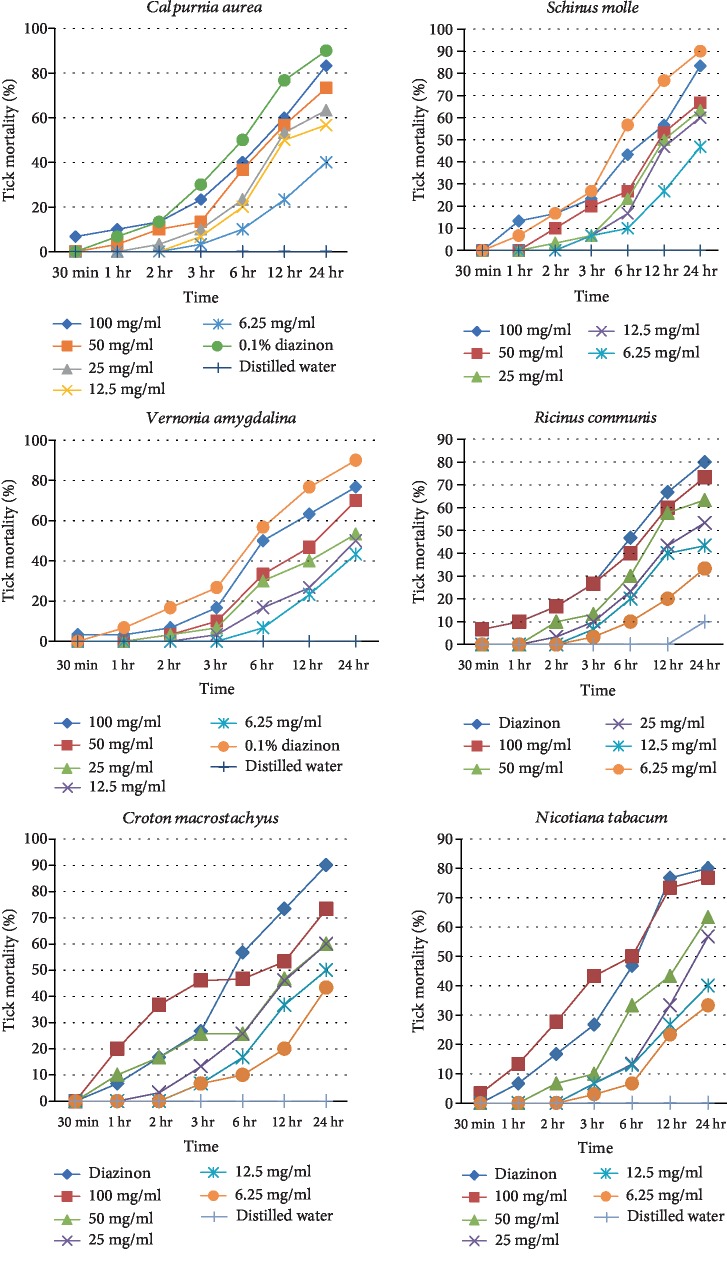
Mortalities of ticks treated with methanolic extracts of selected medicinal plants.

Starting from 3 hr post exposure with 100 mg/ml concentration of *V. amygdalina* leaf extract and diazinon, there showed a significant increase in tick mortality. Starting from 12 hr post exposure, the 6.25 mg/ml concentration of *V. amygdalina* extract has caused significantly higher mortality compared to the distilled water (negative control) (*P* < 0.05). At 24 hr post exposure period, diazinon and 25, 50, and 100 mg/ml concentrations of the extract have caused significantly higher tick mortality than the rest of the concentrations below 12.5 mg/ml (*P* < 0.05) (Figure 1).

A significant increase in tick mortality started 2 hr post exposure with 100 mg/ml concentration of *R. communis* extract and diazinon and 3 hr post exposure with 100 mg/ml concentration of the extract. At 24 hr post exposure period, diazinon and 50 and 100 mg/ml concentrations of the extract have caused significantly higher tick mortality than the rest of the concentrations below 25 mg/ml (*P* < 0.05). The least concentration (6.25 mg/ml) has caused significantly higher mortality when compared with the negative control at 24 hr exposure time (Figure 1).

Tick mortality was significantly increased starting from 2 hr and in 3 hr post exposure of 100 mg/ml concentrations *C. macrostachyus* and diazinon, respectively. At the 24 hr post exposure period, 50 and 100 mg/ml concentrations of the *C. macrostachyus* extract and diazinon had a comparable tick killing effect than the rest of the concentrations below 25 mg/ml (Figure 1).

At the 24 hr post exposure period, diazinon and 50 and 100 mg/ml concentrations of the *N. tabacum* extract have caused significantly higher tick mortality than the rest of the concentrations below 25 mg/ml (*P* < 0.05). However, the three higher concentrations of the extract and diazinon had no significant difference in their effect on the parasite. Moreover, there is no significant difference between the three higher concentrations tested (≥50 mg/ml) (Figure 1).

The mean mortality ± standard errors of mean of *Rh.* (*B*)*. decoloratus* and *Rh. pulchellus* at minute/hour post exposure with different concentrations of *C. aurea*, *S. molle*, *V. amygdalina*, *R. communis*, *C. macrostachyus*, and *N. tabacum* was showed in Tables 3–8. The findings indicated that both *C. aurea* and *S. molle* showed 8.33 ± 0.33 mortality whereas *V. amygdalina*, *R. communis*, *C. macrostachyus*, and *N. tabacum* showed 7.67 ± 0.33, 7.3 ± 0.33, 7.3 ± 0.33, and 7.67 ± 0.33, respectively, mortality effect after 24 hr of exposure to 100 mg/ml concentrations (Tables 3–8).

## 4. Discussion

This study revealed that there was a difference yield percentage of extracts among the plants. The leaf of *R. communis* presented the highest yield (30.67%) among extracts followed by *C. macrostachyus* (29.3%) while the lowest (12%) was observed for the leaf of *V. amygdalina* (Table 1). This finding was in contrary with Askale [11] who reported 9.3% methanolic leaf extract of *R. communis*. Some differences on the percentage yield of these extract materials among the plants might be due to the difference on the nature of plant species. The different results may be also due to chemical composition differences of the extracts, different environmental conditions which create differences in phytochemical constitution, and harvest time. Furthermore, the solvents and test protocols used during extraction promote difference in concentrations and classes of secondary bioactives present in extracts [23].

In the phytochemical screening test, *C. aurea* (leaf) was found positive for alkaloids, saponins, phlobatannin, steroids, flavonoids, glycosides, phenolic compounds, and tannins, while negative for triterpenes (Table 2). This finding is in consistency with Dula and Zelalem [13] who found all the listed active ingredients using different standard tests and Umer et al. [24] who reported the presence of alkaloids, tannins, flavonoids, and saponins. Other study showed that extract of *C. aurea* leaves was found positive for alkaloids [25] and phenolic compounds [26]. The crude extract of *S. molle* (leaf) was positive for tannins and alkaloids while negative for saponins, steroids, flavonoids, glycosides, phlobatannin, phenolic compounds, and triterpenes which is in line with a similar study by Magadum et al. [27] who reported the presence of tannins. This finding was inconsistence with Ribeiro et al. [28] who reported the presence of saponin.

Leaf extracts of *V. amygdalina* contained saponins, tannins, phenolic compound, flavonoids, and alkaloids while negative for steroids, phlobatannins, glycosides, and triterpenes. This finding is similar with Ayoola et al. [29] who reported the presence of saponins and phenolic compounds. Another study by Adebayo et al. [30] also demonstrated that *V. amygdalina* contain tannins, flavonoids, and alkaloids and by Alemu et al. [31] reported the presence of saponin, phenolic compounds, flavonoids, and alkaloids. Phytochemical screening of leaf extracts of *R. communis* revealed that the plant contained saponins, tannins, phenolic compounds, steroids, flavonoids, glycosides, triterpenes, phlobatannin, and alkaloids. The current findings are similar to those reported previously by Singh et al. [32] but different with the findings of Kumar et al. [33] who reported the absence of alkaloids in leaf extracts of *R. communis*. Differences in climatic conditions, tests used, the cultivation, and collection of plant materials for extract production may cause differences in results [34]. Growth conditions of the plants also determine the types and quantities of secondary metabolites derived from natural sources that are subject to variability [35].

The current study revealed that the mean mortality of adult ticks was increased significantly with increased dosage (concentration) and exposure time after *in vitro* treatment for the tested botanicals. This result is in line with the findings of Qwarse [36] and Gebre-Egziabiher [37] in which mortality effect of botanicals was indicated to be dose concentration- and exposure time-dependent. Our study also revealed that all methanolic extracts of the tested botanical leaves at tested concentrations induced significant acaricidal effect against *B. decoloratus* compared with the negative control.

The *in vitro* tick killing activity of methanolic leaf extracts of *C. aurea* is increased with increased concentrations and time exposure (Table 3). After 24 hr post exposure, *C. aurea* showed high mortality (8.33 ± 0.33) at concentrations of 100 mg/ml which was comparable with 0.1% diazinon (positive control) (9.00 ± 0.00). All concentrations of *C. aurea* showed tick killing effects at different concentrations and time exposure compared with the negative control. The present result is comparable with those obtained using different species of ticks reported by some researchers. Fouche et al. [38] reported that the acetone extract of *C. aurea* (leaves and flowers) had mortality effect of 92.2% while the ethanol extract had 81.1% mortality against *R. turanicus.* Similarly, Zorloni et al. [25] reported that 20% and 10% acetone leaf extracts for *C. aurea* either kill or severely compromise the movement of unfed adult *Rh. pulchellus* ticks. Regassa [9] found 10% tick mortality at 5 hr exposure of aqueous leaf and bark extracts of *C. aurea*. Differences among these studies might be due to the difference in solvent used for extraction as studies have shown that organic solvent extracts show greater biological activity than the aqueous extract [39].

Similar to *C. aurea*, *S. molle* showed good *in vitro* tick killing effect. As concentration and exposure time increased, mortality of *Rh. decoloratus* was also increased. At the highest concentration (100 mg/ml), the killing effect of the plant was comparable to the positive control. All concentrations of *S. molle* including the least concentrations had effects on ticks when compared with the negative control (Table 4). In addition, especially for concentrations of 100 mg/ml, 50 mg/ml, and 25 mg/ml, more than 50% of tick mortality was observed as early as 12 hr post exposure to the extracts. Generally, a positive correlation was noted between graded concentrations of the extracts, the exposure test-time interval, and tick mortality (Figure 1). Feyera and Abdisa [12] recorded that the crude aqueous extracts of *S. molle* produced 100% for *Boophilus* adult mortality rates at concentrations of 1 g/25 ml after 24 hr of exposure. Such difference might be due to test solvents used to extract plant and doses applied.

The leaf extracts of *V. amygdalina* showed moderate antitick activity against *B. decoloratus* at higher concentration (100 mg/ml) and have little activity at least concentration (6.25 mg/ml) after 24 hr post exposure. Kumar et al. [33] and Ghosh et al. [40] have reported that similar methanolic extract of the plant has potent activity on *Rh. decoloratus* even at lower concentrations. This finding is incompatible with Askale [11] who found no mortality of *B. decoloratus* with leaf extracts of *V. amygdalina* at 6.25 mg/ml after 24 hr post exposure. The presence of efficacy at low concentration in this finding may be attributed to differences in methodology. It could also be related to the nature of the plant at the time of collection [41]. The acaricidal activity of this plant may be due to the presence of the phytochemical ingredients found in it.

Methanolic extract of *R. communis* leaf had good acaricidal activity (73%) against *Rh. pulchellus* at 100 mg/ml concentration. Kumar et al. [42] had reported that similar methanolic extract of the plant has potent activity on *Rh. pulchellus* even at lower concentrations. It has been reported that the high toxicity of *R. communis* extracts against ticks was due to the presence of bioactive in the extracts [43]. The results obtained in the present study indicated that *C. macrostachyus* and *N. tabacum* had similar acaricidal efficacy on *Rh. pulchellus* with *R. communis*. In line with this finding, Kumar et al. [33] and Ghosh et al. [40] had reported that similar activity of methanolic extract of the plant had shown on *Rh. pulchellus* even at lower concentrations. Different constituents of the plants screened may be responsible for the toxic effect of the extracts that caused mortality of *Rh. pulchellus* [44, 45]. *N. tabacum* was used in the ethnoveterinary practice as an anthelmintic [46], antirheumatic agent, and anti-inflammatory [47]. Phytochemical screening of the various solvent extracts of *N. tabacum* leaves revealed that the plant contains tannins, phenolic compounds, steroids, flavonoids, phlobatannin, and alkaloids.

Comparative *in vitro* acaricidal activity of crude extracts of the plants revealed higher mortality of ticks exposed to *C. aurea* (83%) and *S. molle* (83%) compared with *N. tabacum* (77%), *V. amygdalina* (76%), *R. communis* (73%), and *C. macrostachyus* (73%) after 24 hrs of exposure at 100 mg/ml concentration. The difference in mortality percentage of these plants might be due to variability on the amount of secondary metabolites among the plant extracts. The phytochemical analysis in this study showed that *C. aurea* has more secondary metabolites than others. In this study, the relatively lower acaricidal activity of *N. tabacum*, *V. amygdalina*, *R. communis*, and *C. macrostachyus* compared to *C. aurea* and *S. molle* might be due to lower quantity of secondary metabolites [48]. Studies indicate the presence of alkaloid, glycosides, and phenol as important chemicals to initiate the mechanism of action in *in vitro* and *in vivo* activities causing mortality against ticks [33]. Comparable tick mortality that was obtained by the positive control group (diazinon) might be possible with the studied leaf extract, although there is great variation in time between them. However, due to their cost effectiveness and availability in the rural area, leaves of these plants could be an excellent acaricidal option.

## 5. Conclusions

Extracts of *C. aurea*, *S. molle*, *V. amygdalina*, *R. communis*, *C. macrostachyus*, and *N. tabacum* were tested against *Rh. (B). decoloratus* and *Rh. pulchellus* for their killing efficacy at different concentrations and time intervals. The findings suggest that the crude methanol extracts of the plants contain secondary metabolites with high acaricidal activity properties. It was observed that except *R. communis* and *C. macrostachyus*, which had moderate acaricidal activity, the remaining plants particularly *C. aurea* and *S. molle* had strong acaricidal activity greatly comparable to the effect of 0.1% diazinon at higher concentrations. Efficacy of the extracts increases with increasing concentration and exposure time. Therefore, the present study concluded that the tested medicinal plants showed promising killing effect against *Rh.* (*B*)*. decoloratus* and *Rh. pulchellus* that could be used as the potential alternative to substitute commercially available drugs. For those extracts that have shown high promising results, *in vivo* toxicity and efficacy test should be done to validate the importance of the materials in order to formulate and incubate the products obtained.

## Figures and Tables

**Table 1:** Yield of methanolic crude extracts of all selected medicinal plant leaf.

Plant name	Yield in gram	Yield in %
*C. aurea*	62	20.67
*S. molle*	58	19.33
*V. amygdalina*	36	12
*R. communis*	72	30.67
*C. macrostachyus*	68	29.3
*N. tabacum*	46	22

**Table 2:** Qualitative determinations of active ingredients in crude extracts of different plants.

Ingredients	*C. aurea*	*S. molle*	*V. amygdalina*	*R. communis*	*C. macrostachyus*	*N. tabacum*
Saponin	+	−	+	+	+	−
Tannin	+	+	+	+	+	+
Phenolic compounds	+	−	+	−	+	+
Steroids	+	−	−	−	+	+
Flavonoids	+	−	+	+	−	+
Phlobatannin	+	−	−	+	−	+
Glycosides	+	−	−	+	+	−
Triterpenes	−	−	−	−	−	−
Alkaloids	+	+	+	−	+	+

Note: +: present; −: negative.

**Table 3:** *In vitro* tick killing effect of methanolic extract of *Calpurnia aurea* against *Rh*. (*B*)*. decoloratus*.

Extract concentration (mg/ml)	Mean number of ticks dead (mean of mortality ± SE) at minute/hour post exposure						
	30 min	1 hr	2 hr	3 hr	6 hr	12 hr	24 hr
100	0.67 ± 0.33^b^	1.00 ± 0.00^b^	1.33 ± 0.33^b^	2.33 ± 0.33^bc^	4.00 ± 0.57^de^	6.00 ± 0.57^c^	8.33 ± 0.33^de^
50	0.00 ± 0.00^a^	0.33 ± 0.33^ab^	1.00 ± 0.00^ab^	1.33 ± 0.33^ab^	3.67 ± 0.33^cde^	5.67 ± 0.33^c^	7.33 ± 0.33^cde^
25	0.00 ± 0.00^a^	0.00 ± 0.00^a^	0.33 ± 0.33^ab^	1.00 ± 0.00^ab^	2.33 ± 0.33^bcd^	5.33 ± 0.33^c^	6.33 ± 0.33^bcd^
12.5	0.00 ± 0.00^a^	0.00 ± 0.00^a^	0.00 ± 0.00^ab^	0.67 ± 0.33^a^	2.00 ± 0.00^bc^	5.00 ± 0.00^c^	5.67 ± 0.33^bc^
6.25	0.00 ± 0.00^a^	0.00 ± 0.00^a^	0.00 ± 0.00^a^	0.33 ± 0.33^a^	1.00 ± 0.00^ab^	2.33 ± 0.33^b^	4.33 ± 1.45^b^
0.1% diazinon	0.00 ± 0.00^a^	0.67 ± 0.33^ab^	1.67 ± 0.33^b^	2.67 ± 0.33^c^	5.67 ± 0.33^e^	7.67 ± 0.33^d^	9.00 ± 0.00^e^
Distilled water	0.00 ± 0.00^a^	0.00 ± 0.00^a^	0.00 ± 0.00^a^	0.00 ± 0.00^a^	0.00 ± 0.00^a^	0.00 ± 0.00^a^	0.00 ± 0.00^a^

Means followed by the same letter on the same column are not significantly different by ANOVA (*P* > 0.05).

**Table 4:** *In vitro* tick killing effect of methanolic extract of *Schinus molle* against *Rh.* (*B*)*. decoloratus*.

Extract concentration (mg/ml)	Mean number of ticks dead (mean of mortality ± SE) at minute/hour post exposure						
	30 min	1 hr	2 hr	3 hr	6 hr	12 hr	24 hr
100	0.00 ± 0.00	1.33 ± 0.33^a^	1.67 ± 0.33^b^	2.33 ± 0.33^bc^	4.33 ± 0.33^d^	5.67 ± 0.33^cd^	8.33 ± 0.33^cd^
50	0.00 ± 0.00	0.00 ± 0.00^a^	1.0 ± 0.00^ab^	2.0 ± 0.57^bc^	2.67 ± 0.33^c^	5.33 ± 0.33^c^	6.67 ± 0.33^bc^
25	0.00 ± 0.00	0.00 ± 0.00^a^	0.33 ± 0.33^a^	0.67 ± 0.33^ab^	2.33 ± 0.33^bc^	5.0 ± 0.57^c^	6.33 ± 0.33^bc^
12.5	0.00 ± 0.00	0.00 ± 0.00^a^	0.00 ± 0.00^a^	0.67 ± 0.33^ab^	1.67 ± 0.33^bc^	4.67 ± 0.33^bc^	6.0 ± 0.57^b^
6.25	0.00 ± 0.00	0.00 ± 0.00^a^	0.00 ± 0.00^a^	0.67 ± 0.33^ab^	1.00 ± 0.00^ab^	2.67 ± 0.67^b^	4.67 ± 0.88^b^
0.1% diazinon	0.00 ± 0.00	0.67 ± 0.33^a^	1.67 ± 0.33^b^	2.67 ± 0.33^c^	5.67 ± 0.33^d^	7.67 ± 0.33^d^	9.00 ± 0.00^d^
Distilled water	0.00 ± 0.00	0.00 ± 0.00^a^	0.00 ± 0.00^a^	0.00 ± 0.00^a^	0.00 ± 0.00^a^	0.00 ± 0.00^a^	0.00 ± 0.00^a^

Means followed by the same letter on the same column are not significantly different by ANOVA (*P* > 0.05).

**Table 5:** *In vitro* tick killing effect of methanolic extract of *Vernonia amygdalina* against *Rh*. (*B*)*. decoloratus*.

Extract concentration (mg/ml)	Mean number of ticks dead (mean of mortality ± SE) at minute/hour post exposure						
	30 min	1 hr	2 hr	3 hr	6 hr	12 hr	24 hr
100	0.33 ± 0.33^a^	0.33 ± 0.33^a^	0.67 ± 0.33^ab^	1.67 ± 0.33^cd^	5.00 ± 0.58^cd^	6.33 ± 0.33^de^	7.67 ± 0.33^bc^
50	0.00 ± 0.00^a^	0.00 ± 0.00^a^	0.33 ± 0.33^ab^	1.00 ± 0.00^abc^	3.33 ± 0.33^bc^	4.67 ± 0.33^cd^	7.00 ± 0.58^bc^
25	0.00 ± 0.00^a^	0.00 ± 0.00^a^	0.00 ± 0.00^a^	0.67 ± 0.33^bc^	3.00 ± 0.58^b^	4.00 ± 0.58^bc^	5.33 ± 0.33^b^
12.5	0.00 ± 0.00^a^	0.00 ± 0.00^a^	0.00 ± 0.00^a^	0.33 ± 0.33^ab^	1.67 ± 0.33^ab^	2.67 ± 0.67^bc^	5.00 ± 0.58^b^
6.25	0.00 ± 0.00^a^	0.00 ± 0.00^a^	0.00 ± 0.00^a^	0.00 ± 0.00^a^	0.67 ± 0.33^a^	2.33 ± 0.33^b^	4.33 ± 1.67^b^
0.1% diazinon	0.00 ± 0.00^a^	0.67 ± 0.33^a^	1.67 ± 0.33^b^	2.67 ± 0.33^d^	5.67 ± 0.33^d^	7.67 ± 0.33^e^	9.00 ± 0.00^c^
Distilled water	0.00 ± 0.00^a^	0.00 ± 0.00^a^	0.00 ± 0.00^a^	0.00 ± 0.00^a^	0.00 ± 0.00^a^	0.00 ± 0.00^a^	0.00 ± 0.00^a^

Means followed by the same letter on the same column are not significantly different by ANOVA (*P* > 0.05).

**Table 6:** *In vitro* tick killing effect of methanolic extract of *Ricinus communis* against *Rh. pulchellus*.

Extract concentration (mg/ml)	Mean number of ticks dead (mean of mortality ± SE) at minute/hour post exposure						
	30 min	1 hr	2 hr	3 hr	6 hr	12 hr	24 hr
100	0.67 ± 0.00	1.00 ± 0.33^a^	1.67 ± 0.33^b^	2.67 ± 0.33^b^	4.00 ± 0.57^ab^	6.00 ± 0.57^b^	7.33 ± 0.33^cb^
50	0.00 ± 0.00	0.00 ± 0.00^a^	1.00 ± 0.00^b^	1.33 ± 0.33^ab^	3.00 ± 0.00^c^	5.57 ± 0.33^ab^	6.33 ± 0.33^cb^
25	0.00 ± 0.00	0.00 ± 0.00^a^	0.33 ± 0.33^a^	1.00 ± 0.00^ab^	2.33 ± 0.33^bc^	4.33 ± 0.33^ab^	5.33 ± 0.33^cb^
12.5	0.00 ± 0.00	0.00 ± 0.00^a^	0.00 ± 0.00^a^	0.67 ± 0.33^a^	2.00 ± 0.00^b^	4.00 ± 0.00^b^	4.33 ± 0.33^ab^
6.25	0.00 ± 0.00	0.00 ± 0.00^a^	0.00 ± 0.00^a^	0.33 ± 0.33^a^	1.00 ± 0.00^a^	2.00 ± 0.33^ab^	3.33 ± 1.45^cb^
0.1% diazinon	0.00 ± 0.00	0.67 ± 0.33^a^	1.67 ± 0.33^a^	2.67 ± 0.33^ab^	4.67 ± 0.33^c^	6.67 ± 0.33^ab^	8.00 ± 0.00^cb^
Distilled water	0.00 ± 0.00	0.00 ± 0.00^a^	0.00 ± 0.00^a^	0.00 ± 0.00^a^	0.00 ± 0.00^a^	0.00 ± 0.00^a^	0.00 ± 0.00^a^

Means followed by the same letter on the same column are not significantly different by ANOVA (*P* > 0.05).

**Table 7:** *In vitro* tick killing effect of methanolic extract of *Croton macrostachyus against Rh. pulchellus*.

Extract concentration (mg/ml)	Mean number of ticks dead (mean of mortality ± SE) at minute/hour post exposure						
	30 min	1 hr	2 hr	3 hr	6 hr	12 hr	24 hr
100	0.00 ± 0.00^a^	0.33 ± 0.33^a^	1.33 ± 0.33^b^	1.67 ± 0.33^ab^	2.67 ± 0.33^ab^	5.33 ± 0.33^ab^	7.33 ± 0.33^a^
50	0.00 ± 0.00^a^	0.00 ± 0.00^a^	1.0 ± 0.00^a^	2.0 ± 0.57^ab^	2.57 ± 0.33^b^	4.67 ± 0.33^ab^	6.00 ± 0.33^a^
25	0.00 ± 0.00^a^	0.00 ± 0.00^a^	0.33 ± 0.33^a^	1.33 ± 0.033^ab^	2.57 ± 0.33^b^	4.67 ± 0.57^ab^	6.33 ± 0.33^a^
12.5	0.00 ± 0.00^a^	0.00 ± 0.00^a^	0.00 ± 0.00^a^	0.67 ± 0.33^a^	1.67 ± 0.33^ab^	3.67 ± 0.33^b^	5.0 ± 0.57^a^
6.25	0.00 ± 0.00^a^	0.00 ± 0.00^a^	0.00 ± 0.00^a^	0.67 ± 0.33^a^	1.00 ± 0.00^a^	2.0 ± 0.57^b^	4.33 ± 0.67^a^
0.1% diazinon	0.00 ± 0.00^a^	0.67 ± 0.00^a^	1.67 ± 0.33^a^	2.67 ± 0.0.33^b^	5.67 ± 0.33^ab^	7.33 ± 0.33^ab^	9.00 ± 0.00^a^
Distilled water	0.00 ± 0.00^a^	0.00 ± 0.00^a^	0.00 ± 0.00^a^	0.00 ± 0.00^a^	0.00 ± 0.00^a^	0.00 ± 0.00^a^	0.00 ± 0.00^a^

Means followed by the same letter on the same column are not significantly different by ANOVA (*P* > 0.05).

**Table 8:** *In vitro* tick killing effect of methanolic extract of *Nicotiana tabacum* against *Rh. pulchellus*.

Extract concentration (mg/ml)	Mean number of ticks dead (mean of mortality ± SE) at minute/hour post exposure						
	30 min	1 hr	2 hr	3 hr	6 hr	12 hr	24 hr
100	0.33 ± 0.33	1.33 ± 0.33^a^	2.57 ± 0.33^b^	4.33 ± 0.33^b^	5.0 ± 0.57^ab^	6.33 ± 0.00^ab^	7.67 ± 0.33^ab^
50	0.00 ± 0.00	0.00 ± 0.00^a^	0.67 ± 0.33^ab^	1.00 ± 0.00^ab^	3.33 ± 0.33^ab^	4.33 ± 0.57^ab^	6.33 ± 0.67^ab^
25	0.00 ± 0.00	0.00 ± 0.00^a^	0.00 ± 0.00^a^	0.67 ± 0.33^ab^	1.33 ± 0.33^ab^	3.33 ± 0.57^b^	5.67 ± 0.57^b^
12.5	0.00 ± 0.00	0.00 ± 0.00^a^	0.00 ± 0.00^a^	0.67 ± 0.33^ab^	1.33 ± 0.57^ab^	2.67 ± 0.67^ab^	4.00 ± 0.57^ab^
6.25	0.00 ± 0.00	0.00 ± 0.00^a^	0.00 ± 0.00^a^	0.00 ± 0.00^ab^	0.67 ± 0.33^ab^	2.33 ± 0.33^ab^	3.33 ± 0.33^ab^
0.1% diazinon	0.00 ± 0.00	0.67 ± 0.33^a^	1.67 ± 0.33^ab^	2.67 ± 0.33^ab^	4.67 ± 0.33^b^	7.67 ± 0.33^ab^	8.00 ± 0.00^ab^
Distilled water	0.00 ± 0.00	0.00 ± 0.00^a^	0.00 ± 0.00^a^	0.00 ± 0.00^a^	0.00 ± 0.00^a^	0.00 ± 0.00^a^	0.00 ± 0.00^a^

Means followed by the same letter on the same column are not significantly different by ANOVA (*P* > 0.05).

## Data Availability

Vouchers and dried plant materials used for this study are stored at the Herbarium of the Department of Plant Science, Haramaya University, Ethiopia. The datasets supporting the conclusion of this study are available from the corresponding author on reasonable request.
